# A Gene Cluster for Biosynthesis of Mannosylerythritol Lipids Consisted of 4-*O*-β-D-Mannopyranosyl-(2*R*,3*S*)-Erythritol as the Sugar Moiety in a Basidiomycetous Yeast *Pseudozyma tsukubaensis*

**DOI:** 10.1371/journal.pone.0157858

**Published:** 2016-06-21

**Authors:** Azusa Saika, Hideaki Koike, Tokuma Fukuoka, Shuhei Yamamoto, Takahide Kishimoto, Tomotake Morita

**Affiliations:** 1 Research Institute for Sustainable Chemistry, National Institute of Advanced Industrial Science and Technology (AIST), Tsukuba, Ibaraki, Japan; 2 Bioproduction Research Institute, National Institute of Advanced Industrial Science and Technology (AIST), Tsukuba, Ibaraki, Japan; 3 Toyobo Co., Ltd., Tsuruga Institute of Biotechnology, Tsuruga, Fukui, Japan; Tulane University Health Sciences Center, UNITED STATES

## Abstract

Mannosylerythritol lipids (MELs) belong to the glycolipid biosurfactants and are produced by various fungi. The basidiomycetous yeast *Pseudozyma tsukubaensis* produces diastereomer type of MEL-B, which contains 4-*O*-β-D-mannopyranosyl-(2*R*,3*S*)-erythritol (*R*-form) as the sugar moiety. In this respect it differs from conventional type of MELs, which contain 4-*O*-β-D-mannopyranosyl-(2*S*,3*R*)-erythritol (*S*-form) as the sugar moiety. While the biosynthetic gene cluster for conventional type of MELs has been previously identified in *Ustilago maydis* and *Pseudozyma antarctica*, the genetic basis for MEL biosynthesis in *P*. *tsukubaensis* is unknown. Here, we identified a gene cluster involved in MEL biosynthesis in *P*. *tsukubaensis*. Among these genes, *PtEMT1*, which encodes erythritol/mannose transferase, had greater than 69% identity with homologs from strains in the genera *Ustilago*, *Melanopsichium*, *Sporisorium* and *Pseudozyma*. However, phylogenetic analysis placed PtEMT1p in a separate clade from the other proteins. To investigate the function of *PtEMT1*, we introduced the gene into a *P*. *antarctica* mutant strain, Δ*PaEMT1*, which lacks MEL biosynthesis ability owing to the deletion of *PaEMT1*. Using NMR spectroscopy, we identified the biosynthetic product as MEL-A with altered sugar conformation. These results indicate that PtEMT1p catalyzes the sugar conformation of MELs. This is the first report of a gene cluster for the biosynthesis of diastereomer type of MEL.

## Introduction

**Fig 1:**
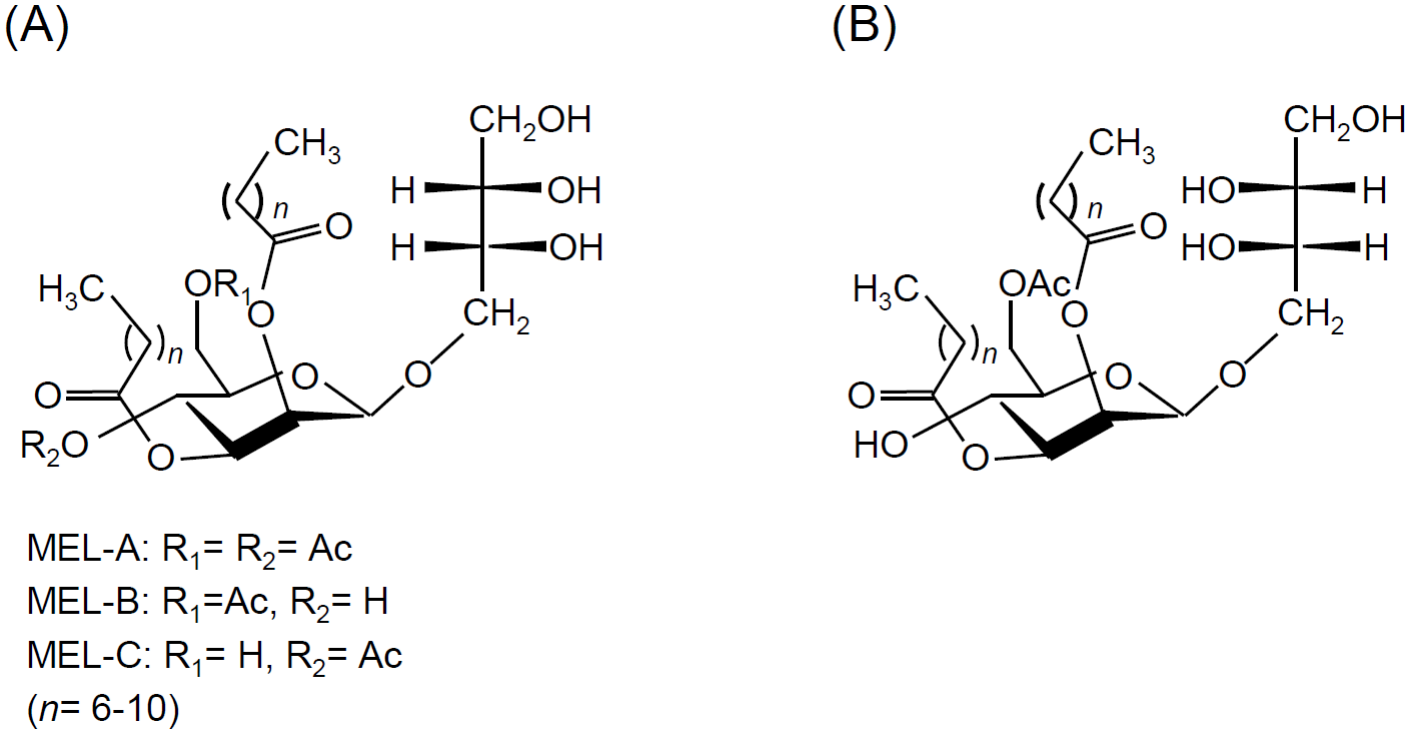
Chemical structure of MELs. (A) Conventional type of MELs. (B) Diastereomer type of MEL-B.

Mannosylerythritol lipids (MELs) belong to the glycolipid biosurfactants which consist of mannosylerythritol (ME) as the hydrophilic moiety, and fatty acids as the hydrophobic moiety. MELs distinguish by conformation of ME. MELs which consist of 4-*O*-β-D-mannopyranosyl-(2*S*,3*R*)-erythritol (*S*-form) is termed conventional type of MELs, and consist of 4-*O*-β-D-mannopyranosyl-(2*R*,3*S*)-erythritol (*R*-form) is termed diastereomer type of MEL (Fig 1). MELs have received a great deal of industrial attention, owing to factors such as their biodegradability, biocompatibility, and favorable interfacial and self-assembling properties [1–3]. MELs have also been used as an ingredient in cosmetics because of their beneficial role in damaged hair and skin repair, cell activation and anti-oxidation [4–7].

MELs are produced from feedstocks by various fungi such as the genera *Ustilago* and *Pseudozyma*. *Ustilago maydis*, a fungal plant pathogen, has been identified as a MEL producer [8, 9] and its complete genome has been sequenced and published [10]. *P*. *antarctica* T-34 (formerly *Candida antarctica* T-34) has also been isolated and found to be an excellent MEL producer, producing more than 100 g L^-1^ of MELs [11]. Similarly, several other *Pseudozyma* species have been established as MEL producers [12, 13]. Recently, genome sequences of species from the genus *Pseudozyma* have been reported. These species include *P*. *antarctica* T-34 and JCM10317[14, 15], *P*. *aphidis* DSM70725 [16] and *P*. *hubeiensis* SY62 [17]. Gene expression vectors and transformation methods have also been developed for this genus [18–20]. Owing to their high productivity and the availability of gene manipulation techniques [21], these *Pseudozyma* species are promising candidates for the commercial production of MELs.

Of the yeast species in the genus *Pseudozyma*, *P*. *antarctica*, *P*. *parantarctica*, *P*. *aphidis* and *P*. *rugulosa* produce mainly MEL-A (more than 70% of all MELs produced) [11, 12, 22–26]. *P*. *graminicola*, *P*. *hubeiensis*, *P*. *siamensis* and *P*. *shanxiensis* primarily produce MEL-C [27–31], and various strains of *P*. *tsukubaensis* produce the diastereomer type of MEL-B (Fig 1B) in large quantities [32]. The physicochemical properties of these molecules depend on their chemical structure, including the acetylation pattern, the conformation of fatty acids and the sugar moiety. The hydrophilicity of MEL-A (di-acetylated MEL) is lower than those of MEL-B and MEL-C (mono-acetylated MELs). Also, the water-holding property of *R*-form ME in diastereomer type of MELs is higher than that of *S*-form ME [33]. Our previous reports showed that diastereomer type of MEL-B has self-assembling properties across a wide range of concentrations and temperatures [34] and shows higher hydration ability than conventional type of MELs [35]. Therefore, diastereomer type of MEL-B produced by *P*. *tsukubaensis* may facilitate the use of MELs in aqueous solutions.

**Fig 2:**
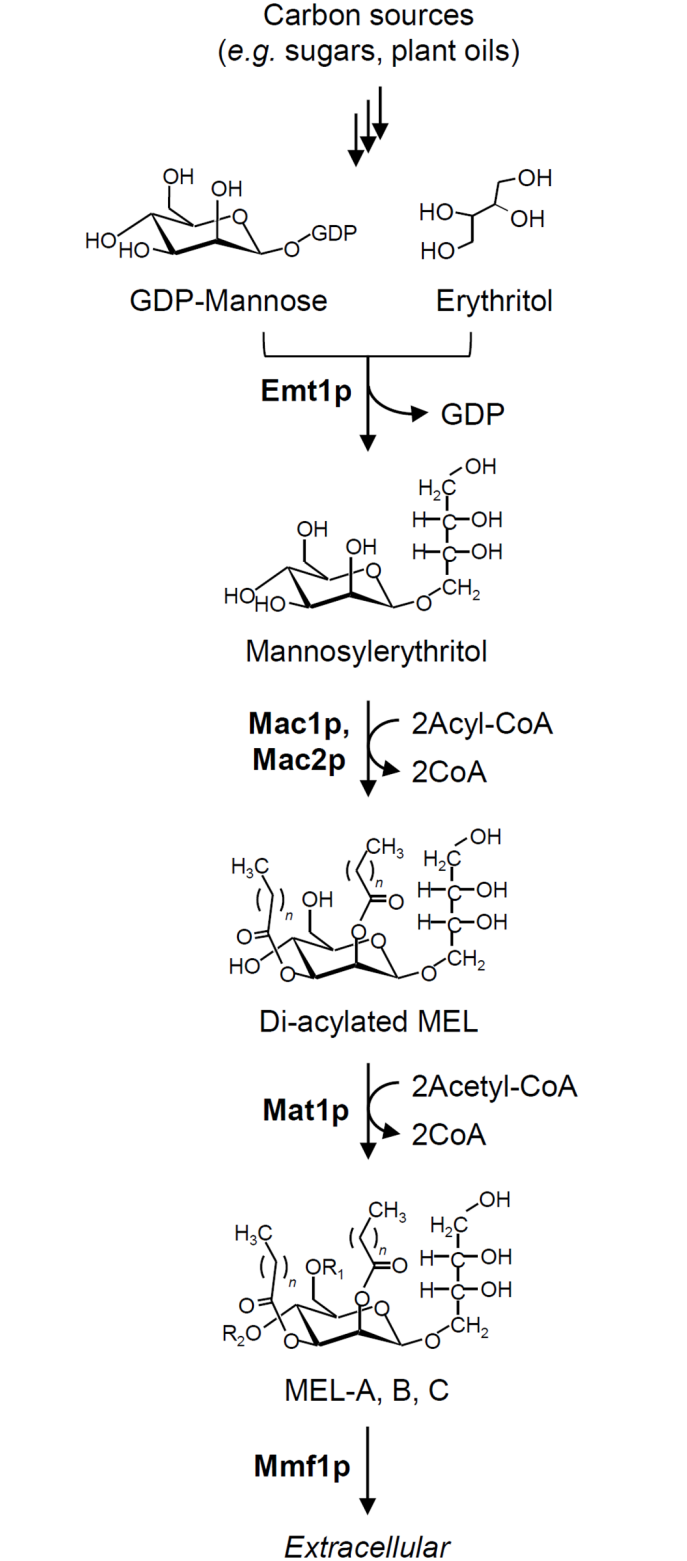
The biosynthetic pathway of MELs. Emt1p: erythritol/mannose transferase. Mac1p and Mac2p: acyl transferases. Mat1p: acetyl transferase. Mmf1p: putative transporter.

Over the past decade, a gene cluster involved in the biosynthesis of various glycolipids (such as MELs, cellobiose lipids and sophorolipids) has been identified [36–40]. In *U*. *maydis*, the MEL biosynthetic pathway consists of five proteins: an erythritol/mannose transferase (Emt1p), two acyl transferases (Mac1p and Mac2p), an acetyltransferase (Mat1p) [36, 37] and a putative transporter (Mmf1p) (Fig 2). While the products of *P*. *antarctica* are similar to those of *U*. *maydis*, the sugar conformation of MEL produced by *P*. *tsukubaensis* differs from those of the other *Ustilago* and *Pseudozyma* species. Furthermore, the degree of acetylation of the mannose moiety in *P*. *tsukubaensis* differs from that of *P*. *antarctica*, because the main product of *P*. *tsukubaensis* is a monoacetylated MEL (MEL-B). Therefore, the reaction of MEL biosynthesis in *P*. *tsukubaensis* differs from the reactions in *P*. *antarctica* and *U*. *maydis*, particularly with respect to sugar conformation and acetylation. We therefore focused the current study on the gene cluster for MEL biosynthesis in *P*. *tsukubaensis*.

We identified the gene cluster responsible for biosynthesis of the diastereomer type of MEL-B in *P*. *tsukubaensis* NBRC1940 based on amino acid sequence analysis. The putative amino acid sequence encoded by the gene *PtEMT1* exhibited high identity to that of *P*. *antarctica*, but had an independent position on the phylogenic tree. We altered the sugar conformation of MELs in *P*. *antarctica* from *S*-form to *R*-form by introducing *PtEMT1* from *P*. *tsukubaensis* into a gene-disrupted mutant of *P*. *antarctica* lacking MEL biosynthesis.

## Materials and Methods

### *Pseudozyma* strains and plasmid

*Pseudozyma tsukubaensis* NBRC1940 was purchased from NITE Biological Resource Center (NBRC; Tokyo, Japan). A gene-disrupted mutant of *P*. *antarctica* T-34, Δ*PaEMT1*, is our laboratory stock [21]. A *PtEMT1* expression vector, pUXV1_neo::PtEMT1, and *PaEMT1* expression vector, PUXV1_neo::PaEMT1 were introduced to a host strain, Δ*PaEMT1*, by electroporation [19], resulting in a strain complemented the lacking of MEL biosynthesis ability.

### Sequence analysis

The draft genome sequence of *P*. *tsukubaensis* NBRC1940 was performed (reported elsewhere). The BLAST program was used for sequence similarity searching in a database available on the NCBI website (http://blast.ncbi.nlm.nih.gov/Blast.cgi). Multiple sequence alignments were displayed using the ClustalW program. Phylogenetic analysis was performed using the neighbor-joining method [41] with the program MEGA6 [42] and bootstrap analysis based on 1,000 replicates [43].

### Plasmid construction

**Fig 3:**
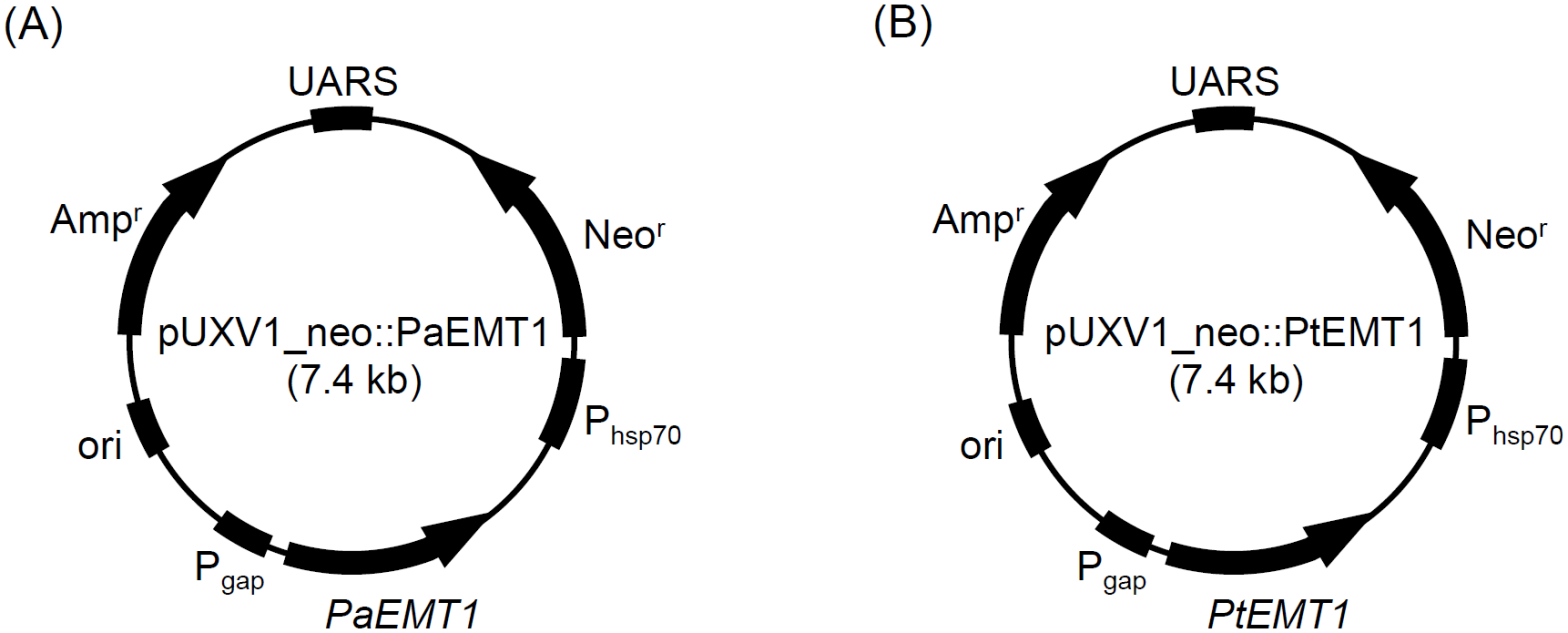
The plasmid maps of pUXV1_neo::PaEMT1 and pUXV1_neo::PtEMT1.

The plasmid pUXV1_neo::PaEMT1 (Fig 3A) was constructed in Morita et al. (2013) [44]. The plasmid pUXV1_neo::PtEMT1 (Fig 3B) was constructed as follows. A *PtEMT1* fragment was amplified with BamHI site by PCR using the complementary DNA of *P*. *tsukubaensis* NBRC1940 as template, and a set of oligonucleotide primers: 5’- GTTTGGATCCATGAAAGTGGCACTGCTTTC-3’ (forward), and 5’-CGGGATCCCATGAGGGAACTGATGTGCG-3’ (reverse). The 1.8-kb *PtEMT1* fragment was digested by BamHI, and then inserted at the corresponding site in pUXV1_neo, yielding the plasmid pUXV1_neo::PtEMT1.

### Transformation

The plasmid pUXV1_neo::PtEMT1 and pUXV1_neo::PaEMT1 were introduced into Δ*PaEMT1* by electroporation according to Morita *et al*. [19] with suitable modification. Δ*PaEMT1* was grown in 3 mL MEL production medium (1 g L^-1^ of yeast extract, 3 g L^-1^ of NaNO_3_, 0.3 g L^-1^ of KH_2_PO_4_ and 0.3 g L^-1^ of MgSO_4_·7H_2_O) containing 10% (w/v) glycerol at 25°C for 3 days as a seed culture. One milliliter of seed culture was inoculated into 50 mL of MEL production medium containing 10% (w/v) glycerol and cultivated at 25°C for 15 h with 250 stroke min^-1^. The cells were harvested by centrifugation at 5,000 rpm for 5 min and washed twice with chilled 1 M sorbitol. 0.1 mL of cell suspension containing about 3 μg of plasmid was pulsed by electroporation using the Bio-Rad Gene Pulser II with Pulse Controller Plus (Bio-Rad, Tokyo, Japan). The cells were pulsed twice with a square-wave electroporation pulse of 1000 V and a pulse length of 1.0 ms at a pulse interval of 5 s. The electroporated cells were immediately diluted in 0.9 mL of chilled 1 M sorbitol and 3 mL of YM medium and incubated at 25°C for 1 h. After incubation, an aliquot (0.2 mL) was spread on the YM medium plate containing 500 μg mL^-1^ G418, and the resulting colonies were grown at 25°C for 4 days.

### MEL production

Δ*PaEMT1* harboring pUVX1_neo::PtEMT1 or pUXV1_neo::PaEMT1 were cultivated in 2 mL YM medium (3 g L^-1^ of yeast extract, 3 g L^-1^ of malt extract, 5 g L^-1^ peptone and 10 g L^-1^ glucose) containing 200 μg mL^-1^ G418 at 25°C for 3 days as a seed culture. In a 300-mL flask, the seed culture was inoculated with 30 mL MEL production medium containing 10% (w/v) glucose as a carbon source and incubated at 25°C for 7 days with 200 stroke min^-1^. 200 μg mL^-1^ of G418 was supplied in the culture medium to maintain the plasmid. The produced MELs were detected using TLC [19]. The MELs were extracted from the cell culture with an equal volume of ethyl acetate, and 50 μL of ethyl acetate extracts were analyzed by TLC using chloroform, methanol and NH_4_OH in a 65:15:2 (v:v:v) ratio as an eluent. The MELs were detected on the TLC plate by spraying with 2% anthrone-sulfate reagent and heating at 90°C for 5 min. A mixture of purified MEL-A, MEL-B and MEL-C was used as a reference.

### MEL purification

The ethyl acetate fractions containing MELs were evaporated. The concentrated MELs were dissolved in chloroform and purified using silica gel (Wako-gel C-200) column chromatography with a gradient elution of chloroform/acetone (10:0 to 0:10, v/v) mixtures as solvent systems [23]. The purified MEL-A was used in the following experiments.

### Structural analysis

The structure of the purified MEL-A was characterized by ^1^H and ^13^C nuclear magnetic resonance spectroscopy (NMR) with a Bruker AVANCE 400 (400 MHz) at 30°C in a CDCl_3_ solution. Tetramethylsilane [(CH_3_)_4_Si] was used as an internal chemical shift standard and the purified MEL-A was used as a reference for NMR analysis.

## Results

### Identification of the gene cluster of MEL biosynthesis in *P*. *tsukubaensis* NBRC1940

**Fig 4:**
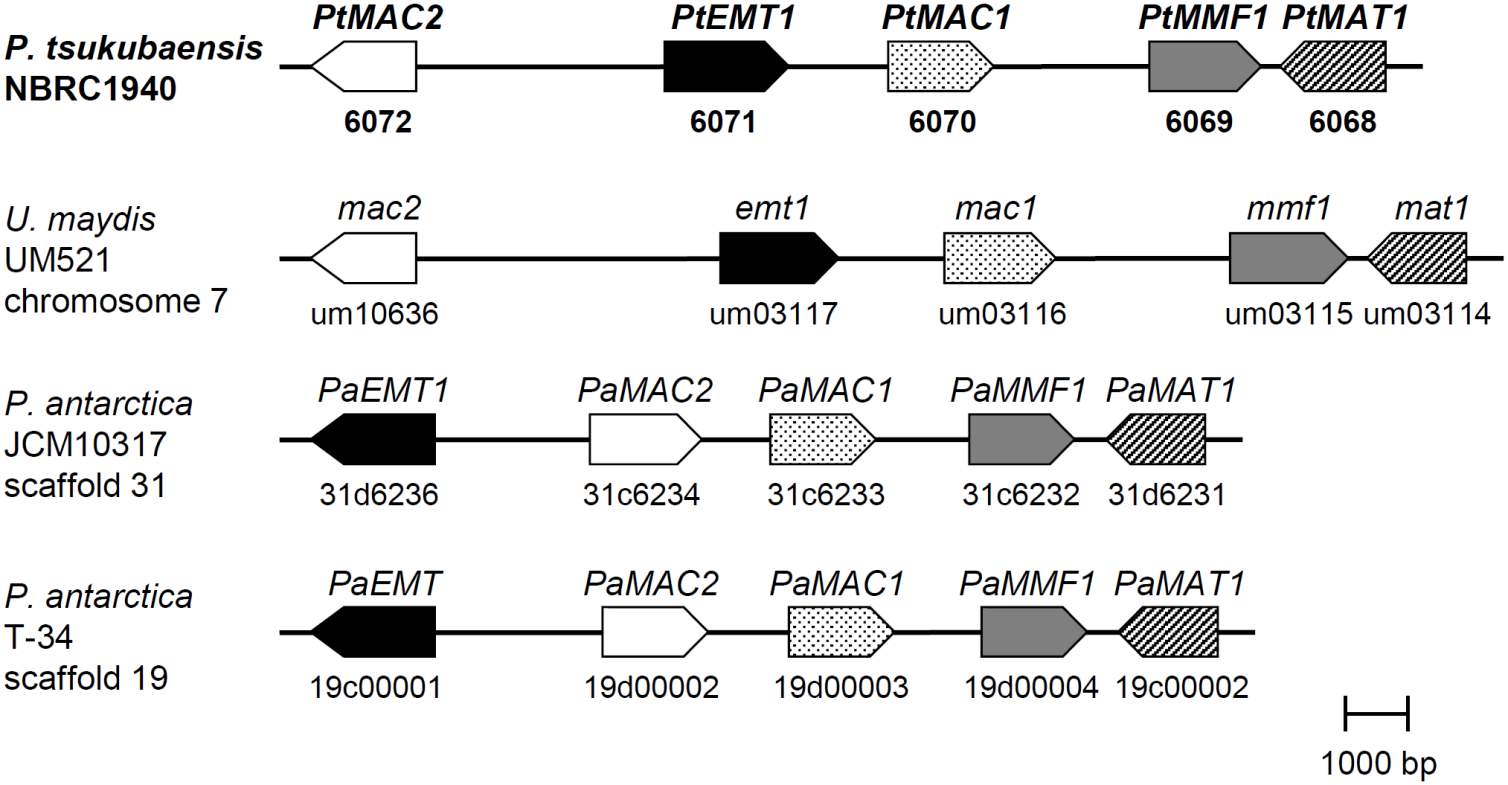
Gene clusters of MEL biosynthesis. Emt1: erythritol/mannose transferase; Mac1 and Mac2: acyl transferases; Mat1: acetyl transferase; Mmf1: putative transporter.

Based on BLASTP analyses, the genes involved in MEL biosynthesis (*emt1*, *mac1*, *mac2*, *mmf1* and *mat1* for *U*. *maydis*; *PaEMT1*, *PaMAC1*, *PaMAC2*, *PaMMF1* and *PaMAT1* for *P*. *antarctica*) were found in the draft genome of *P*. *tsukubaensis* NBRC1940 (*PtEMT1*, *PtMAC1*, *PtMAC2*, *PtMMF1* and *PtMAT1*, respectively) (Fig 4). The gene arrangement in the MEL biosynthesis cluster of *P*. *tsukubaensis* NBRC1940 is more similar to *U*. *maydis* than *P*. *antarctica* strains JCM10317 and T-34 (Fig 4). The *PtEMT1* and *PtMAC2* of *P*. *tsukubaensis* NBRC1940 are rearranged, as well as *U*. *maydis*, compared with *P*. *antarctica* JCM10317 and T-34.

The amino acid sequence homology of five proteins involved in the biosynthesis of MELs (PtEMT1p, PtMAC1p, PtMAC2p, PtMMF1p and PtMAT1p) was compared to those from *U*. *maydis* and *P*. *antarctica* strains JCM10317 and T-34 (Table 1). According to BLASTP analysis, PtEMT1p, PtMAC1p, PtMAC2p and PtMMF1p have high (over 50%) homology to the corresponding proteins in *U*. *maydis* and *P*. *antarctica*.

**Table 1:** The amino acid sequence identities of proteins in the MEL biosynthesis gene cluster compared to *U*. *maydis* and *P*. *antarctica*.

Proteins	Size (aa)	Functions	Identity (%)^a^		
			*U*. *maydis* UM521	*P*. *antarctica* JCM10317^T^	*P*. *antarctica* T-34
PtEMT1p	612	Erythritol/mannose transferase	69	69	70
PtMAC1p	557	Acyl transferase	60	64	63
PtMAC2p	549	Acyl transferase	60	57	57
PtMMF1p	584	Putative transporter	71	75	75
PtMAT1p	543	Acetyl transferase	55	52	53

^a^ Analyzed by BLASTP.

### Amino acid sequence analysis of *PtEMT1* from *P*. *tsukubaensis* NBRC1940

The amino acid sequence of PtEMT1p is similar to those of the corresponding proteins in *U*. *maydis* and *P*. *antarctica*. However, the sugar conformation of MEL produced by *P*. *tsukubaensis* differed from those of other MEL producers. We thus performed further amino acid sequence analysis (Table 2). PtEMT1p consists of 612 amino acids, and the corresponding proteins from *U*. *maydis* UM521, *P*. *antarctica* JCM10317 and *P*. *antarctica* T-34 consist of 615, 617 and 617 amino acids, respectively. Surprisingly, only nine proteins, from strains in the genera *Ustilago*, *Melanopsichium*, *Sporisorium* and *Pseudozyma*, showed high sequence identities with PtEMT1p (69–72%) (Table 2). All of these strains, except for those in the genus *Pseudozyma*, have been reported as plant pathogens [9, 45, 46]. Table 3 shows Emt1p sequence identities among *P*. *tsukubaensis* NBRC1940 and nine other strains. While PtEMT1p has approximately 70% identity with homologous proteins, other pairs of species (e.g. *P*. *aphidis* and *P*. *antarctica* or *S*. *scitamineum* and *S*. *reilianum*) share more than 90% sequence identity.

**Table 2:** The homologous proteins of PtEMT1p by BLASTP analysis.

#	Accession^a^	Description^a^	Strains^a^	e-value^b^	Identity (%)^b^
1	CDR99457.1	hypothetical protein	*Sporisorium scitamineum*	0	72
2	CCF52717	uncharacterized protein UHOR_04876	*Ustilago hordei*	0	70
3	CDI53946	conserved hypothetical protein	*Melanopsichium pennsylvanicum* 4	0	70
4	ETS61959	mannosyltransferase	*Pseudozyma aphidis* DSM 70725	0	69
5	GAC96558	glycosyltransferase	*Pseudozyma hubeiensis* SY62	0	70
6	GAK68006	glycosyltransferase	*Pseudozyma antarctica*	0	69
7	GAC75887	hypothetical protein PANT_19c00001	*Pseudozyma antarctica* T-34	0	70
8	CBQ73522	conserved hypothetical protein	*Sporisorium reilianum* SRZ2	0	70
9	XP_011389468	erythritol-mannosyl-transferase involved in MEL production	*Ustilago maydis* 521	0	69
10	BAI77915	mannosyltransferase	*Pseudozyma antarctica*	0	68
11	XP_006666634	putative glycosyltransferase	*Cordyceps militaris* CM01	5E-101	37
12	XP_681404	hypothetical protein AN8135.2	*Aspergillus nidulans* FGSC A4	4.62E-94	35
13	XP_008597660	mannosyltransferase-like protein	*Beauveria bassiana* ARSEF 2860	9.3E-92	35
14	KHN93740	UDP-glucuronosyl/UDP-glucosyltransferase	*Metarhizium album* ARSEF 1941	1.13E-86	39
15	KGQ12602	putative UDP-glucuronosyltransferase ugt-47	*Beauveria bassiana* D1-5	3.8E-65	38
16	KIM30075	glycosyltransferase family 1 protein	*Serendipita vermifera* MAFF 305830	6.33E-23	24
17	CCU99492	unnamed protein product	*Malassezia sympodialis* ATCC 42132	4.33E-19	37
18	XP_001730757	hypothetical protein MGL_1756	*Malassezia globosa* CBS 7966	3.22E-18	35
19	XP_001728828	hypothetical protein MGL_3995	*Malassezia globosa* CBS 7966	1.47E-17	36
20	KIM75119	glycosyltransferase family 1 protein	*Piloderma croceum* F 1598	1.4E-15	47

^a^ Referred from NCBI database.

^b^ Analyzed by BLASTP.

**Table 3:** Comparison of amino acid sequence homology of PtEMT1p and homologous proteins.

Subject sequence	Identity (%)^a^									
	Query sequence									
	P. tsu	S. sci	U. hor	M. pen	P. aph	P. hub	P. ant	P. ant T-34	S. rei	U. may
P. tsu	-	72	70	70	69	70	69	70	71	69
S. sci	72	-	80	82	77	81	78	77	93	82
U. hor	70	80	-	78	75	77	76	76	79	78
M. pen	70	82	79	-	78	80	78	77	82	79
P. aph	69	77	76	78	-	77	97	94	78	75
P. hub	70	81	77	80	77	-	78	77	80	82
P. ant	69	78	77	78	97	78	-	95	78	76
P. ant T-34	70	77	77	77	94	77	95	-	77	75
S. rei	70	92	77	81	77	80	78	77	-	80
U. may	69	82	78	79	75	82	76	75	81	-

^a^ Analyzed by BLASTP.

P. tsu, *Pseudozyma tsukubaensis* NBRC1940; S. sci, CDR99457.1_*Sporisorium scitamineum*; U. hor, CCF52717_*Ustilago hordei*; M. pen, CDI53946_*Melanopsichium pennsylvanicum* 4; P. aph, ETS61959_*Pseudozyma aphidis* DSM70725; P. hub, GAC96558_*Pseudozyma hubeiensis* SY62; P. ant, GAK68006_*Pseudozyma antarctica*; P. ant, GAC75887_*Pseudozyma antarctica* T-34; S. rei, CDR99457.1_*Sporisorium reilianum* SRZ2; U. may, XP_011389468_*Ustilago maydis* 521.

**Fig 5:**
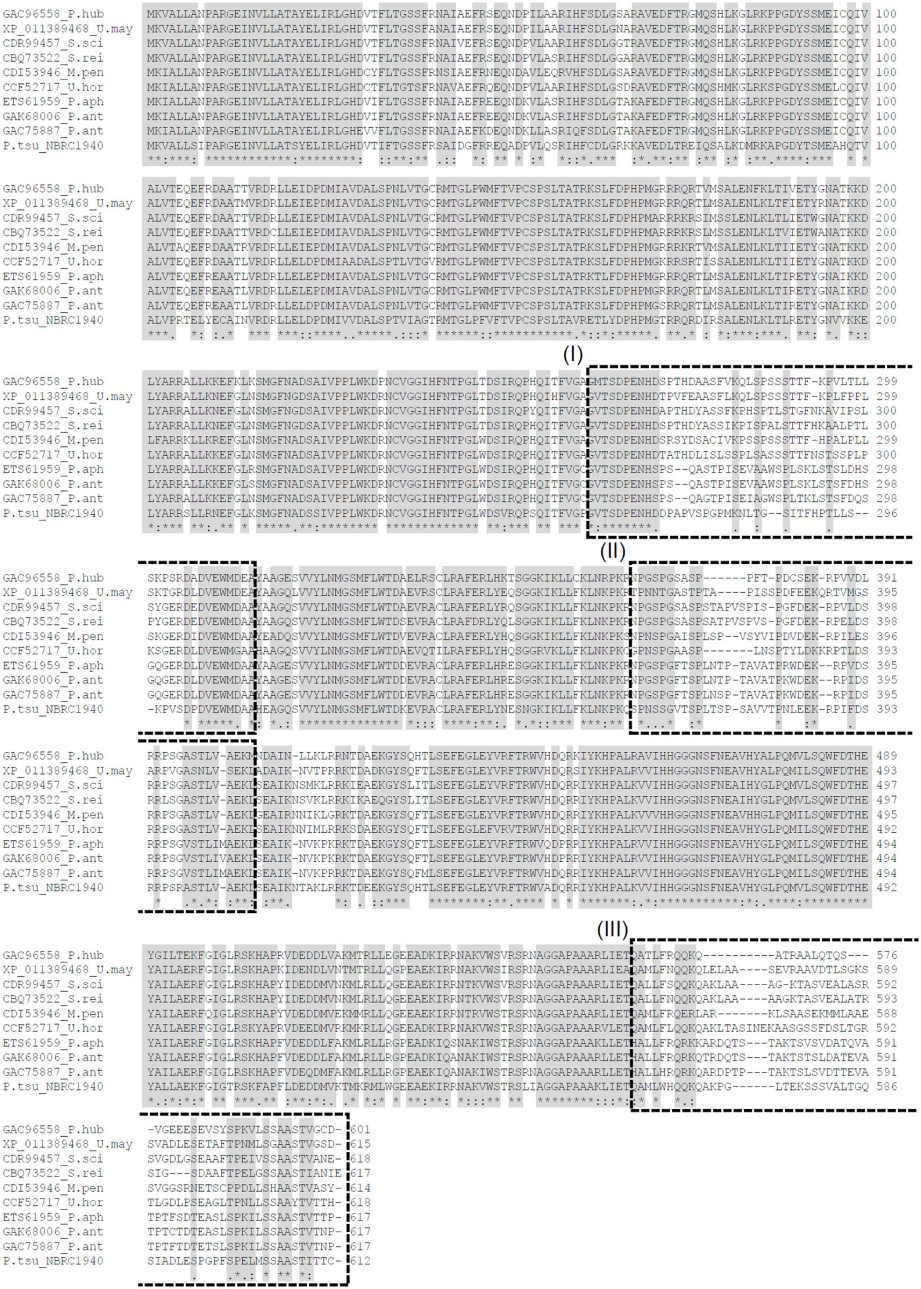
Amino acid sequence alignment of the PtEMT1p from *P*. *tsukubaensis* NBRC1940 and nine homologous proteins. (I), (II) and (III) show low-identity regions I, II, and III, respectively. Identical residues are shown on a black background. GAC96558_P. hub: *Pseudozyma hubeiensis* SY62. XP_011389468_U. may: *Ustilago maydis* 521. CDR99457.1_S. sci: *Sporisorium scitamineum*. CBQ73522_S. rei: *Sporisorium reilianum* SRZ2. CDI53946_M. pen: *Melanopsichium pennsylvanicum* 4. CCF52717_U. hor: *Ustilago hordei*. ETS61959_P. aph: *Pseudozyma aphidis* DSM70725. GAK68006_P. ant: *Pseudozyma antarctica*. GAC75887_P. ant: *Pseudozyma antarctica* T-34. P. tsu: *Pseudozyma tsukubaensis* NBRC1940.

**Fig 6:**
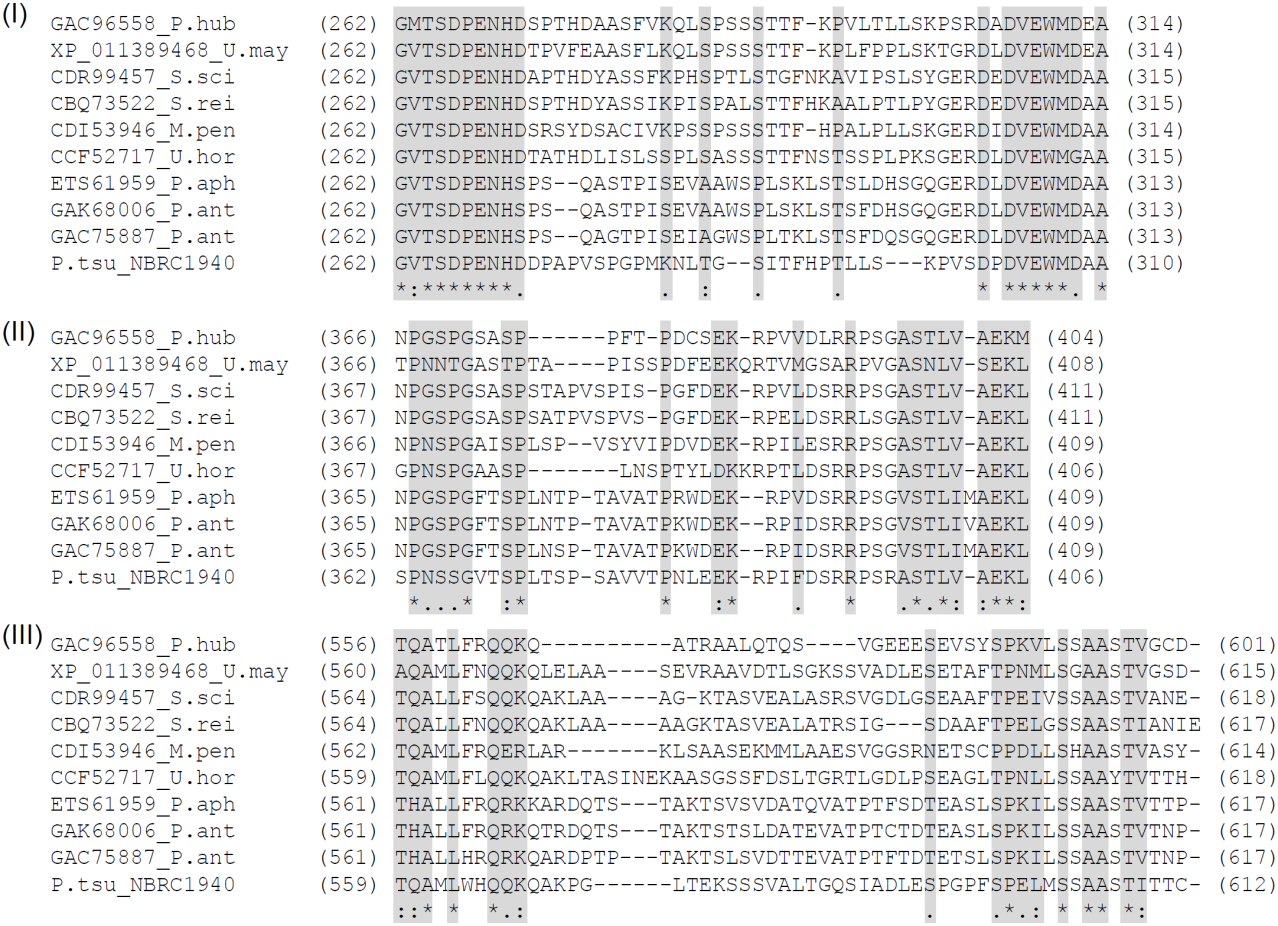
Enlarged view of regions I, II and III.

**Fig 7:**
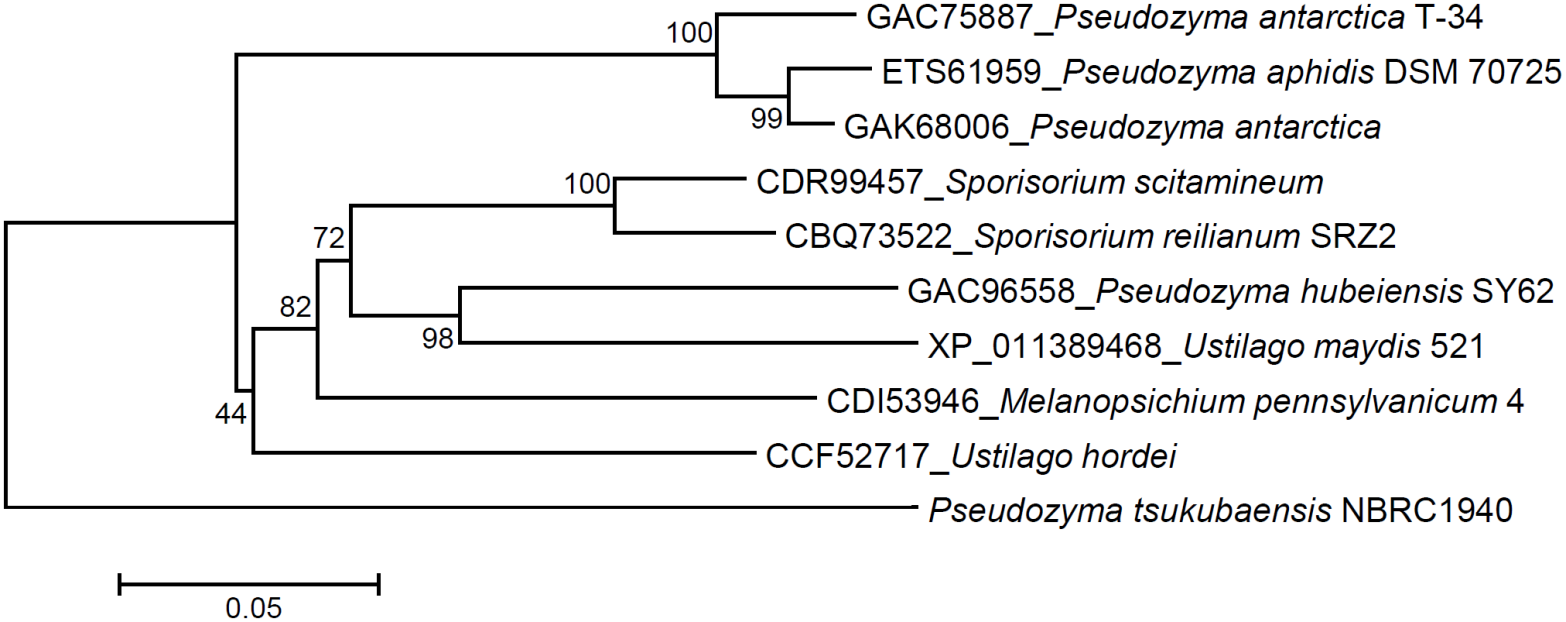
The phylogenetic relationships among *P*. *tsukubaensis* NBRC1940 and homologous strains, based on the Emt1p amino acid sequence.

We aligned nine proteins that exhibited high identity to PtEMT1p using ClustalW. While sequence identity was greater than 69%, we found three regions with relatively low identity (Fig 5). The three regions in *P*. *tsukubaensis* NBRC1940 each consisted of about 30 amino acids. The amino acid position of regions I, II and III were from 272 to 300, 372 to 397, and 569 to 597, respectively (Fig 6). Putative sugar binding domain was not found. Phylogenetic analysis based on the amino acid sequences of Emt1p indicated that PtEMT1p diverged significantly from the other nine proteins (Fig 7). Thus, amino acid sequence analysis suggests that PtEMT1p is a novel structure that differs substantially from homologous proteins.

### MEL production by *P*. *antarctica* Δ*PaEMT1* harboring pUXV1_neo::PtEMT1 from P. *tsukubaensis* NBRC1940

Because Δ*PaEMT1* derived from *P*. *antarctica* T-34 had its *PaEMT1* function disrupted, it provides a useful host to investigate the function of *PtEMT1* in MEL production. The plasmid harboring *PtEMT1* from *P*. *tsukubaensis* NBRC1940 was introduced into Δ*PaEMT1*. The empty vector pUXV1_neo was used as a negative control, and pUXV1_neo::PaEMT1 which harboring *PaEMT1* from *P*. *antarctica* T-34 was used as a positive control.

**Fig 8:**
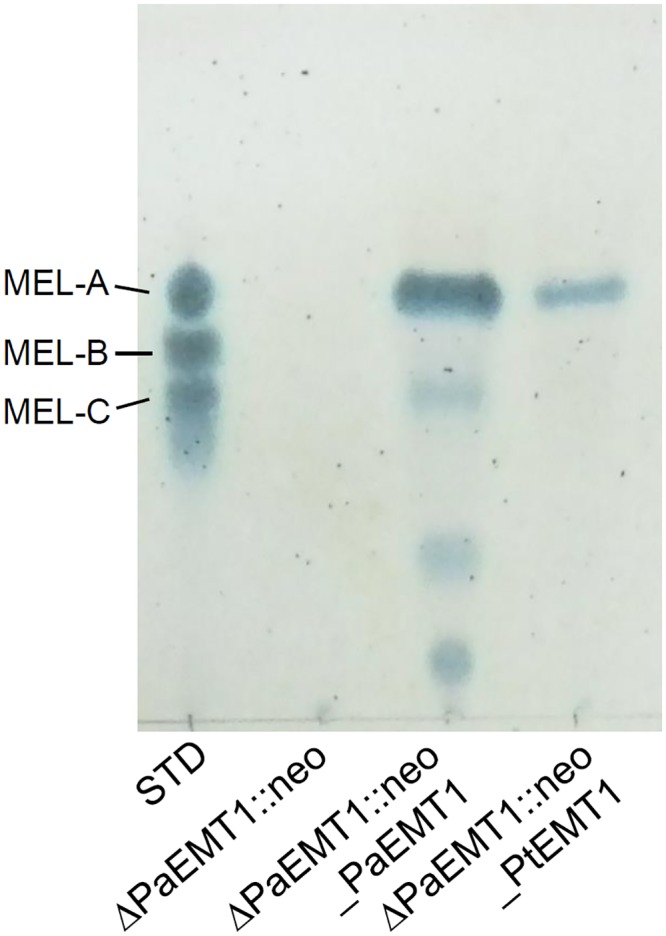
TLC analysis of MEL production by Δ*PaEMT1* harboring pUXV1_neo::PtEMT1. STD: standard MELs containing MEL-A, MEL-B and MEL-C. ΔPaEMT1::neo: Δ*PaEMT1* harboring pUXV1_neo. ΔPaEMT1::neo_PaEMT1: Δ*PaEMT1* harboring pUXV1_neo::PaEMT1. ΔPaEMT1::neo_PtEMT1: Δ*PaEMT1* harboring pUXV1_neo::PtEMT1. Each strain was cultivated in MEL production medium containing 10% (w/v) glucose for 7 days at 25°C. The spots were visualized using anthrone reagent.

The Δ*PaEMT1* harboring pUXV1_neo::PtEMT1 was cultivated in MEL production medium containing 10% (w/v) glucose for 7 days at 25°C. The produced MELs were extracted by ethyl acetate and detected by thin-layer chromatography (TLC) analysis (Fig 8). Δ*PaEMT1* harboring pUXV1_neo::PtEMT1 and pUXV1_neo::PaEMT1 produced MELs from glucose, whereas Δ*PaEMT1* harboring empty vector PUVX1_neo failed to produce MELs, as expected. Thus, the gene *PtEMT1* from *P*. *tsukubaensis* NBRC1940 restored MEL production in Δ*PaEMT1*.

### Structural analysis of diastereomer type of MEL-A biosynthesized by Δ*PaEMT1* harboring pUXV1_neo::PtEMT1

**Fig 9:**
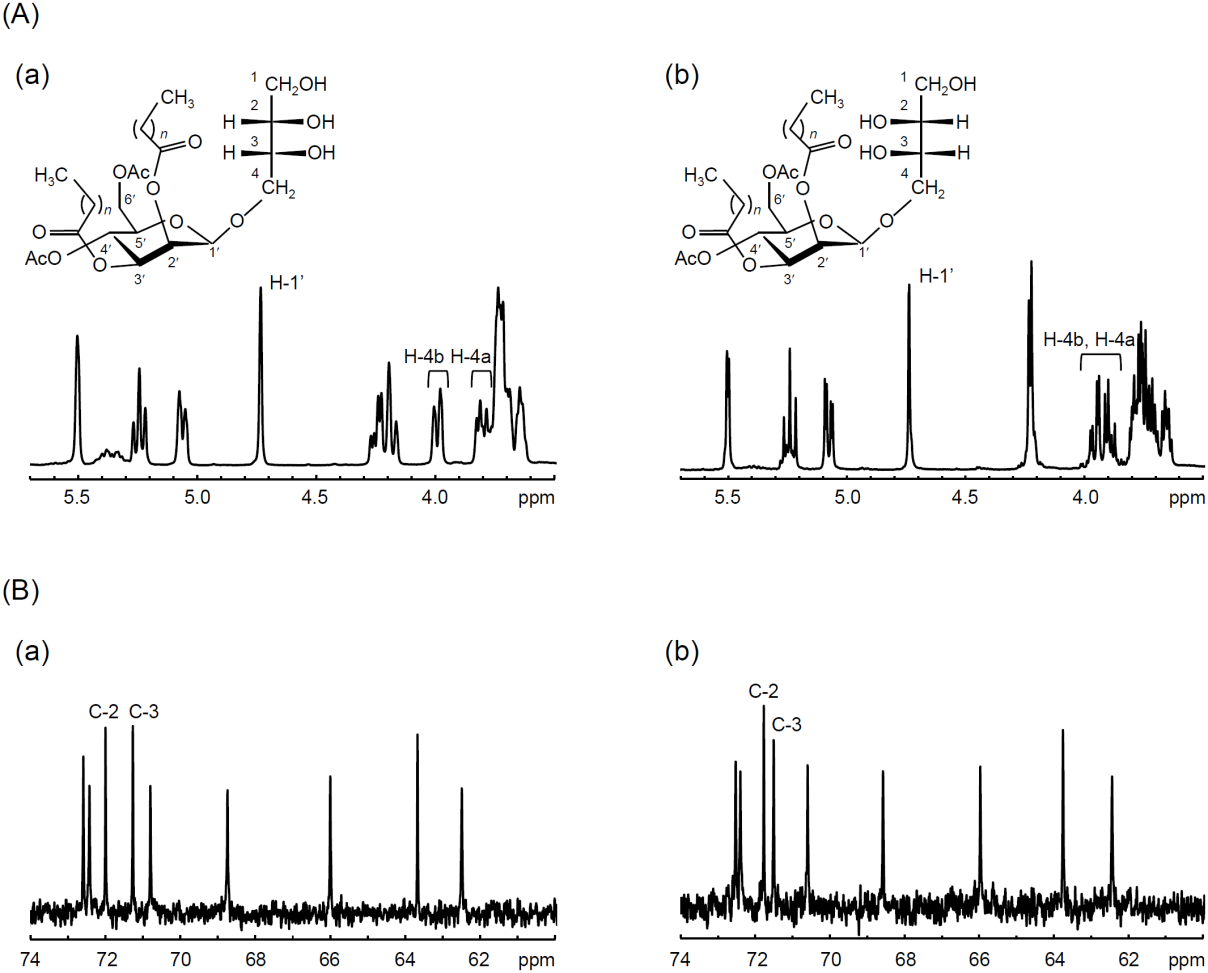
Partial ^1^H and ^13^C NMR spectra. (A) ^1^H NMR spectra. (B) ^13^C NMR spectra. (a): conventional type of MEL-A synthesized by Δ*PaEMT1* harboring pUXV1_neo::PaEMT1, (b): MEL-A synthesized by Δ*PaEMT1* harboring pUXV1_neo::PtEMT1.

To determine the structure of MEL-A produced by Δ*PaEMT1* expressing *PtEMT1*, we subjected purified MEL-A to NMR analysis and compared the signal pattern to that of conventional type of MEL-A produced by Δ*PaEMT1* expressing *PaEMT1* (Fig 9A and 9B). Each signal was assigned as previously described [47–49]. The diastereomer and conventional type of MELs showed very similar ^1^H NMR spectra (Fig 9A). However, two resonances arising from H-4a and H-4b in the erythritol moiety were significantly different. In conventional type of MEL the H-4 signals were widely separated (H-4b: 3.98–4.01 ppm; H-4a: 3.80–3.83 ppm), while the signals observed from diastereomer type of MEL overlapped with one another (H-4a and H4-b: 3.87–3.97 ppm). Moreover, the H-1’ signal from the mannose anomeric hydrogen was shifted to a lower field in MEL-A produced by recombinant strain Δ*PaEMT1* harboring pUXV1_neo::PtEMT1 (from 4.73 ppm to 4.74 ppm). In ^13^C NMR analyses (Fig 9B), we observed a characteristic chemical shift in the C-2 and C-3 signals of the erythritol moiety (from 72.0 ppm to 71.8 ppm and 71.3 ppm to 71.5 ppm, respectively), which corresponded to the C-2 and C-3 signals in diastereomer type of MEL-A [47–49]. Based on these observations, we concluded that Δ*PaEMT1* harboring pUXV1_neo::PtEMT1 produced the diastereomer type of MEL-A.

## Discussion

In this study, we obtained for the first time the gene cluster involved in the diastereomer type of MEL-B biosynthesis in *P*. *tsukubaensis*. The gene *PtEMT1*, which plays a crucial role in determining the sugar conformation of ME, was introduced into Δ*PaEMT1*. *PtEMT1* restored MEL production in Δ*PaEMT1* and the product was determined to be diastereomer type of MEL-A.

MEL biosynthesis clusters have been previously reported in conventional type of MEL producers such as *U*. *maydis*, *P*. *antarctica*, *P*. *aphidis* and *P*. *hubeiensis* [14–17, 36, 37]. Earlier studies assessed the function of *Emt1* using gene disruption methods and found that *Emt1* is essential for MEL biosynthesis [21, 36]. Therefore, we strongly expected *Emt1* to contribute to the chirality of MEL-B in *P*. *tsukubaensis*. To investigate this hypothesis, we introduced the plasmid pUXV1_neo::PtEMT1 into Δ*PaEMT1*, which lacks MEL production. NMR analyses revealed that the MEL produced was diastereomer type of MEL-A (Fig 9). We therefore concluded that PtEMT1p plays a critical role in the formation of the sugar moiety in MELs.

Morita *et al*. [50] reported that *P*. *tsukubaensis* JCM16987 synthesizes mannosyl-L-arabitol lipid-B from L-arabitol as a substrate but does not utilize D-arabitol. In contrast, *P*. *parantarctica*, a conventional type of MEL-A producer, biosynthesized only mannosyl-D-mannitol lipid from D-arabitol. This suggests that substrate specificity in *P*. *tsukubaensis* differs from that of conventional type of MEL producers [50]. Based on Emt1p amino acid sequence alignment (Fig 5), we observed three low-identity regions in the C-terminal half of *P*. *tsukubaensis* NBRC1940. We predict that these regions may be related to the substrate specificity of sugar alcohols in *P*. *tsukubaensis*. Important avenues for future research, include chimeric enzyme construction, crystal structure analysis, and determination of the active site of PtEMT1p, will provide us with greater insight into the function of PtEMT1p.

To our knowledge, *P*. *tsukubaensis* and *P*. *crassa* are the only species that biosynthesize diastereomer type of MEL [48, 49]. *P*. *crassa* biosynthesizes a mixture of diastereomer type of MELs containing MEL-A, MEL-B and MEL-C; however, the genomics of this species has not yet been studied. Therefore, genetic and structural analysis of Emt1p in *P*. *crassa* will help to elucidate the catalytic mechanism of sugar conformation.

Various homologs of conventional type of MELs have been reported to date, such as MEL-A, MEL-B, MEL-C and MEL-D. Other MEL homologs, containing D-arabitol, D-mannitol and ribitol instead of erythritol, have also been biosynthesized [51, 52]. In previous studies, diastereomer type of MEL-B and MEL-D exhibited higher hydrophilicity and water-holding properties than conventional type of MELs [35]. Based on these findings, it was determined that the sugar conformation of ME affects the function of MELs. Hitherto, MEL-B is the only diastereomer type of MEL capable of commercial-scale production. Diastereomer type of MEL-A and MEL-C are biosynthesized by *P*. *crassa*; however, this species produces only small amounts of MELs. While diastereomer type of MEL-D can be obtained *in vitro* by enzymatic reaction, there is no previously reported means of producing this glycolipid through microbial biosynthesis. In the present study, we demonstrated that diastereomer type of MEL-A can be produced by a conventional type of MEL-A producer modified to express *PtEMT1*. Further investigation of the PtEMT1p enzyme will facilitate the expansion of structural and functional varieties of MELs through gene engineering methods.

In conclusion, we identified for the first time the gene cluster involved in diastereomer type of MEL-B biosynthesis and demonstrated that PtEMT1p plays a crucial role in sugar conformation. Further elucidation of the MEL biosynthesis gene cluster will provide opportunities for metabolic engineering using this biosynthetic pathway.
